# Glucagon-like Peptide 1 Receptor Agonists in Cardio-Oncology: Pathophysiology of Cardiometabolic Outcomes in Cancer Patients

**DOI:** 10.3390/ijms252011299

**Published:** 2024-10-21

**Authors:** Vincenzo Quagliariello, Maria Laura Canale, Irma Bisceglia, Martina Iovine, Vienna Giordano, Ilaria Giacobbe, Marino Scherillo, Domenico Gabrielli, Carlo Maurea, Matteo Barbato, Alessandro Inno, Massimiliano Berretta, Andrea Tedeschi, Stefano Oliva, Alessandra Greco, Nicola Maurea

**Affiliations:** 1 Division of Cardiology, Istituto Nazionale Tumori-IRCCS-Fondazione G. Pascale, 80131 Napoli, Italy; martina.iovine@istitutotumori.na.it (M.I.); vienna.giordano@istitutotumori.na.it (V.G.); ilaria.giacobbe@istitutotumori.na.it (I.G.); matteo.barbato@istitutotumori.na.it (M.B.); n.maurea@istitutotumori.na.it (N.M.); 2 U.O.C. Cardiologia, Ospedale Versilia, 55041 Lido di Camaiore, Italy; marialaura.canale@uslnordovest.toscana.it; 3 Servizi Cardiologici Integrati, Dipartimento Cardio-Toraco-Vascolare, Azienda Ospedaliera San Camillo Forlanini, 00148 Rome, Italy; irmabisceglia@gmail.com; 4 Division of Cardiology, Hospital San Pio Benevento (BN), 82100 Benevento, Italy; marino.scherillo@libero.it; 5 U.O.C. Cardiologia, Dipartimento Cardio-Toraco-Vascolare, Azienda Ospedaliera San Camillo Forlanini, 00152 Rome, Italy; dgabrielli@scamilloforlanini.rm.it; 6 Department of Medicine, University of Salerno, 84084 Fisciano, Italy; carlo.maurea@libero.it; 7 Medical Oncology, IRCCS Ospedale Sacro Cuore Don Calabria, 37024 Negrar di Valpolicella, Italy; alessandro.inno@sacrocuore.it; 8 Department of Clinical and Experimental Medicine, University of Messina, 98122 Messina, Italy; berrettama@gmail.com; 9 Cardiology Unit of Emergency Department, Guglielmo da Saliceto Hospital, 29121 Piacenza, Italy; andrea.tedeschimd@gmail.com; 10 UOSD Cardiologia di Interesse Oncologico IRCCS Istituto Tumori “Giovanni Paolo II”, 70124 Bari, Italy; s.oliva@oncologico.bari.it; 11 Divisione di Cardiologia, Fondazione IRCCS San Matteo Hospital, Viale Golgi 19, 27100 Pavia, Italy; a.greco@smatteo.pv.it

**Keywords:** cardio-oncology, GLP-1 receptor, cardioprotection, cancer, inflammation, metabolism

## Abstract

Cancer patients, especially long cancer survivors, are exposed to several cardio-metabolic diseases, including diabetes, heart failure, and atherosclerosis, which increase their risk of cardiovascular mortality. Therapy with glucagon-like peptide 1 (GLP1) receptor agonists demonstrated several beneficial cardiovascular effects, including atherosclerosis and heart failure prevention. Cardiovascular outcome trials (CVOTs) suggest that GLP-1 RA could exert cardiorenal benefits and systemic anti-inflammatory effects in patients with type-2 diabetes through the activation of cAMP and PI3K/AkT pathways and the inhibition of NLRP-3 and MyD88. In this narrative review, we highlight the biochemical properties of GLP-1 RA through a deep analysis of the clinical and preclinical evidence of the primary prevention of cardiomyopathies. The overall picture of this review encourages the study of GLP-1 RA in cancer patients with type-2 diabetes, as a potential primary prevention strategy against heart failure and atherosclerosis.

## 1. Introduction

Glucagon-like peptide-1 receptor agonists (GLP1-RA) are primarily used for type-2 diabetes [T2DM] and obesity therapy [1,2]. These drugs act by mimicking GLP-1, a G-protein-coupled receptor expressed in several tissues, including cardiomyocytes, the central nervous system, the liver, and others, and also able to stimulate insulin secretion in response to food intake; moreover, GLP-1 is able to reduce glucagon release from pancreatic α cells, promoting satiety, gluconeogenesis, and glycogenolysis in the liver [3]. In recent years, GLP-1 RA has demonstrated several cardio-renal benefits in multiple preclinical and clinical models, including obesity, inflammation, and cardiovascular diseases, even in non-diabetic conditions [4]. The clinically available GLP-1 RA drugs include exenatide (Byetta, Bydureon), liraglutide (Victoza, Saxenda), dulaglutide (Trulicity), semaglutide (Ozempic, Rybelsus), and lixisenatide (Adlyxin) [5,6,7,8]. Notably, all GLP-1 RA drugs have demonstrated an efficient prevention of rapid postprandial spikes in blood glucose levels [9,10,11,12,13] and a significant reduction in visceral obesity, a key player in cardiovascular diseases and breast, prostate, ovary, and liver cancers [14,15].

**Figure 1:**
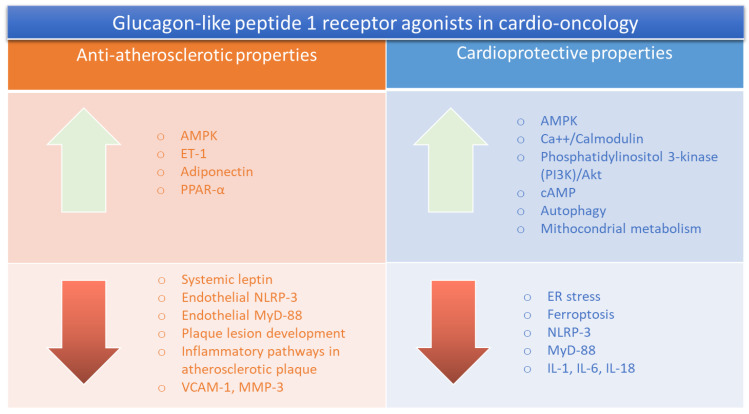
Overall picture of anti-antherosclerotic and cardioprotective properties of GLP-1 RA in cardio-oncology.

A recent study highlighted the direct and indirect effects of GLP-1 RA on the cardiovascular system [16]; in fact, they can improve endothelial function and reduce blood pressure with associated anti-inflammatory effects, contributing to reducing the risk of major adverse cardiovascular events [MACE] [17,18]. Another recent study evidenced how GLP-1 RA was able to reduce albuminuria and the progression of diabetic kidney diseases [19]. From a molecular point of view, as illustrated in Figure 1, GLP-1 RA acts in human cells through cyclic AMP (cAMP) and PI3K/Akt pathways [20]. In more detail, after binding to the GLP-1 receptor, GLP1 RA activates adenylate cyclase, which increases cAMP levels [21]. Notably, cAMP activates protein kinase A [PKA], a key mediator of the intracellular effects of GLP1-RA on pancreatic cells [22]. Activation of the phosphatidylinositol 3-kinase [PI3K]/Akt pathway is another important mechanism of the GLP-1 RA-mediated insulinotropic and cytoprotective effects on pancreatic β cells and liver and cancer cells [23]. Considering the key role of cAMP and PI3K/Akt pathways in cardio-renal diseases, obesity, and T2DM, GLP-1 RA could be a potential pharmacological tool in a wide range of patients, including those with cancer and associated cardiovascular diseases [24,25].

Moreover, GLP1-RA is able to activate AMPK in endothelial and cardiac cells, improving mitochondrial metabolism and autophagy [26] (Figure 1); anti-inflammatory effects are mediated by the inhibition of the NLRP-3 and MyD-88 pathways, leading to increased adiponectin levels and reduced systemic and myocardial IL-1, IL-6, and IL-8 concentrations [27,28]. Therefore, GLP1-RA could be an important tool to reduce the development of cardiovascular diseases and systemic inflammation in cancer patients.

## 2. Biochemical Properties of GLP-1 RA: An Overview

Several large cardiovascular outcome trials with novel glucose-lowering agents, namely, SGLT2i (SGLT2 inhibitors) and GLP-1 RA, have demonstrated robust and significant reductions in major adverse cardiovascular events and additional cardiovascular outcomes, such as hospitalizations for heart failure [29,30,31]. Recent studies have highlighted the anti-atherosclerotic properties of GLP1-RA, through reductions in VCAM-1 and MMP-3 and the enhancement of AMPK and endothelin-1 levels in endothelial cells [32,33]. As reported in the introduction, the most known effect of GLP1-RA is the enhancement of glucose homeostasis in patients with and without type-2 diabetes, independently from the insulin pathways [33,34]. Notably, the reduction in glucose levels contributes to reduced visceral fat and white adipose tissue (WAT), and to an increase in brown adipose tissue (BAT) [35,36], leading to reduced cardio-metabolic and atherosclerotic risk [37,38]. Moreover, GLP-1 RA exerts beneficial renal properties due to a mild diuretic effect based on its stimulation of glucose and sodium excretion [39]. In fact, recent trials have shown that GLP-1 RA can reduce the risk of MACE in patients with type 2 diabetes and the progression of kidney diseases through a reduction in intraglomerular pressure, promoting natriuresis [40,41].

As specified above, GLP-1 RA exerts multiple biochemical effects in several tissues, including renal, cardiac, adipose, and liver [42]. Patients with T2DM are characterized by rapid fat oxidation in heart tissue [43], with a reduced P/O ratio and reduced cardiac work efficacy [44]. In fact, high levels of interleukin-1, 6, and 18 were found in these patients, leading to heart failure [45]. GLP-1 RA reduces fat oxidation and increases glucose oxidation in myocardial tissue, restoring the AMPK, cAMP, and PI3K/Akt levels [46]. Moreover, GLP1-RA is able to reduce the intracellular levels of NLRP-3, MyD-88, and pro-inflammatory cytokines, including IL-1β, IL-6, and growth factors [46,47,48,49]. Other mechanisms of GLP-1 RA-associated cardioprotection include oxidative stress and lipid peroxidation [50]. In more detail, GLP-1 RA reduces intracellular Ca^++^ overload and reactive oxygen species (iROS) in cardiomyocytes, improving mitochondrial functions and reducing lipid peroxidation, preventing ferroptosis [51,52]. Lipid peroxidation is one of the major mechanisms of anthracycline-induced cardiotoxicity, leading to high intracellular levels of MDA and 4-HNA [53,54,55].

**Figure 2:**
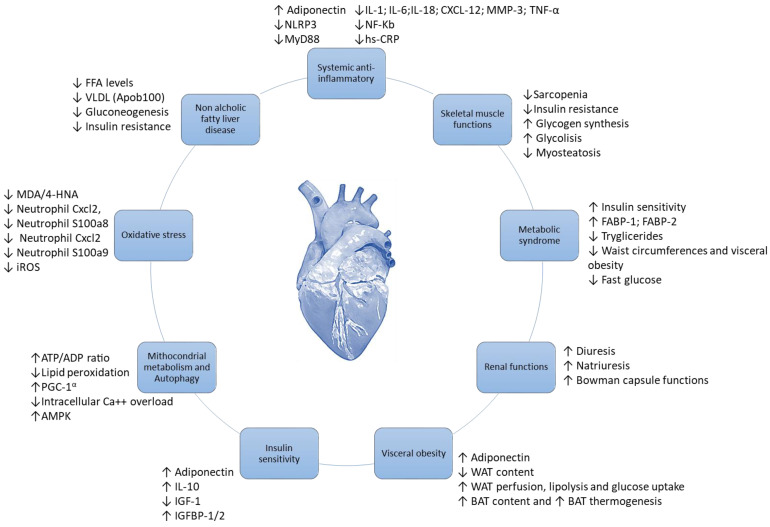
Cardioprotective mechanisms of GLP1-RA against anticancer treatment–induced cardiotoxicity.

As summarized in Figure 2, GLP-1 RA increased pAMPK levels in myocardial and renal tissues, leading to increased mitochondrial functions and a reduction in NLRP-3 expression [56]. The activation of pAMPK and downregulation of NLRP-3 reduces systemic and cardiac leptin, IL-1α, IL-1β, IL-2, IL-4, IL-6, IL17-α, IFN-γ, TNF-α, G-CSF and GM-CSF levels [57]. Moreover, significant increases in IL-10 and adiponectin levels during therapy with GLP-1 RA were also seen, leading to increased autophagy and ATP/ADP ratio in cardiac and renal tissues [58].

The biochemical effects of GLP1-RA improve ejection fraction, fractional shortening and left ventricular thickness. Recent studies indicate beneficial effects of GLP-1 RA on EF also in patients with heart failure with reduced ejection fraction (HFrEF) and type 2 diabetes [14,15]. Another study showed that GLP-1 RA liraglutide and exenatide can improve left ventricular ejection fraction (LVEF) in patients with heart failure, due to their anti-inflammatory, anti-fibrotic and anti-oxidant effects leading to an improvement of endothelial functions and glucose uptake by myocardial cells [56]; these effects may enhance systolic function, particularly in patients with comorbid heart failure and diabetes. Moreover, studies in preclinical models suggest that GLP-1 RA may improve mitochondrial metabolism in myocardial tissues during heart failure and diabetes [57]. Moreover, GLP-1 RA reduced hypertrophy in preclinical and clinical studies, through anti-inflammatory, anti-oxidant and anti-fibrotic effects [57]. In humans, GLP-1 RA reduced left ventricular mass and improved cardiac geometry in patients with diabetic cardiomyopathy or hypertension [57]; these effects are particularly significant considering that hypertrophy and increased wall thickness are common in patients with metabolic syndrome, diabetes, and heart failure with preserved ejection fraction (HFpEF) [23,31,57].

Notably, as specified in Figure 2, GLP-1 RA also exerts indirect beneficial effects in myocardial tissues, through the prevention and treatment of visceral obesity, insulin resistance, NAFLD and T2DM [59]. In more detail, GLP-1RA induces a WAT to BAT switch with an M1-M2 polarization in the adipose microenvironment [60]. Brown Adipose Tissues, rich in adipocytes with M2-polarized macrophages and T-reg cells, change their metabolic functions to a Th1/Th17 polarization phenotype in patients with metabolic syndrome, atherosclerosis and visceral obesity [61]. GLP-1 RA decreased WAT, and reduced associated M-1 macrophages and Th1/Th17 T cells [62]. Interestingly, inflamed WAT was associated with adypokine dysfunctions, involving high systemic levels of leptin and low levels of adiponectin and IL-10, thereby exposing patients to a high risk of insulin resistance and metabolic syndrome [63,64].

## 3. Clinical Evidences of Beneficial Cardiovascular Properties of GLP-1 RA

Table 1 shows a comprehensive overview of seven clinical trials aimed at studying the efficacy and safety of GLP-1 RA in patients with type 2 diabetes, obesity and cardiovascular diseases. These trials explore different dosages, patient populations and clinical outcomes to assess the potential use of GLP1-RA as a key therapeutic intervention [65].

### 3.1. The LEADER Trial

In the LEADER (Liraglutide Effect and Action in Diabetes: Evaluation of Cardiovascular Outcome Results) trial, the authors demonstrated that liraglutide can significantly reduce the risk of MACE, which includes non-fatal myocardial infarction, non-fatal stroke, and cardiovascular death [66]. In more detail, the LEADER trial is a pivotal study that assessed the cardiovascular outcomes of liraglutide in patients with type-2 diabetes at high cardiovascular risk [67]. As a primary outcome, the authors concluded that liraglutide significantly reduced the risk of MACE compared to placebo; however, no significant differences were seen in hospitalization for HF among patients treated with liraglutide [68]. Moreover, the LEADER trial showed that liraglutide also exerts renal benefits, including a reduction in the onset or worsening of nephropathy [69]. The most common side effects included nausea, vomiting and diarrhea. A potential risk of pancreatitis was also seen, but the incidence was very low [70]. Moreover, liraglutide was associated with a low risk of hypoglycemia when used alone or combined with metformin; however, the incidence was increased when combined with sulfonylureas or insulin [71]. Moreover, this trial suggested that liraglutide could be useful in patients with heart failure and HFpEF [72,73,74,75]. In line with these results, the European Society of Cardiology guidelines and the American Diabetes Association have recommended the use of GLP-1 RA in patients with type 2 diabetes and established atherosclerotic cardiovascular diseases [76] (Table 1).

### 3.2. The HARMONY Outcomes Trial

The HARMONY Outcomes trial was designed to evaluate the cardiovascular effects of the GLP-1 RA albiglutide [77]. The primary aim of the HARMONY Outcomes trial was the assessment of cardiovascular safety and efficacy of albiglutide in patients with type 2 diabetes and a high risk of cardiovascular events. In more detail, it was a multicenter, randomized, double-blind, placebo-controlled trial that enrolled 9463 patients [78] randomly assigned to receive either albiglutide (30 mg or 50 mg) or placebo. The primary endpoint was the composite of MACE. The HARMONY Outcomes trial demonstrated that albiglutide reduced the risk of MACE by 22% compared to placebo. Moreover, albiglutide improved hyperglycemia and reduced body weight in enrolled patients [79]. Albiglutide was generally well tolerated, showing a safety profile comparable to placebo. The incidence of severe hypoglycemia and other serious adverse events was very low [79]. In conclusion, the HARMONY Outcomes trial demonstrated for the first time that albiglutide exerted cardiovascular benefits in patients with type-2 diabetes and at high risk of cardiovascular events.

### 3.3. The EXSCEL Trial

The EXSCEL (Examining Cardiovascular Outcomes with Exenatide Extended Release) trial was a major study that sought to evaluate the cardiovascular safety and efficacy of exenatide extended-release (ER), a GLP-1 RA, in patients with type-2 diabetes [80]. In more detail, it was a multicenter, randomized, double-blind, placebo-controlled trial that enrolled 14752 patients with type-2 diabetes with a history of cardiovascular disease or at high risk for cardiovascular events. Participants were randomly assigned to receive either exenatide ER (2 mg once weekly) or placebo and followed for 3.2 years. The primary endpoint was the composite of MACE [81]. In brief, the EXSCEL trial demonstrated that exenatide ER did not significantly reduce the risk of MACE compared to placebo. The hazard ratio for MACE was 0.91, indicating a 9% reduction, but this result was not statistically significant (*p* = 0.17). Also in this case, exenatide ER was associated with improvements in glycemic control and weight reduction with a good tolerability. The safety profile, including the incidence of gastrointestinal events and hypoglycemia, was comparable to placebo [82] (Table 1).

### 3.4. The SUSTAIN Trials

The SUSTAIN (Semaglutide Unabated Sustainability in Treatment of Type 2 Diabetes) clinical trial program included several studies aimed as assessing the efficacy and safety of semaglutide for therapy of type-2 diabetes and associated cardiovascular risk [83]. One of the most important trials in this program is the SUSTAIN-6 trial [84]. This was a multicenter, randomized, double-blind, placebo-controlled trial that involved 3297 patients with type-2 diabetes and established cardiovascular diseases, chronic kidney diseases, or both. In brief, patients were randomized to receive either semaglutide (0.5 mg or 1.0 mg) or placebo once weekly in addition to standard care. The primary composite endpoint was the first occurrence of MACE. The secondary endpoint involves the individual components of the primary composite endpoint; other outcomes involve all-cause mortality, revascularization procedures and hospitalizations for heart failure. In this trial, semaglutide reduced the risk of the primary composite endpoint by 26% compared to placebo (hazard ratio [HR] 0.74; 95% CI 0.58–0.95; *p* < 0.001 for non-inferiority and *p* = 0.02 for superiority). This reduction was associated with a decrease in the incidence of non-fatal stroke (HR 0.61; 95% CI 0.38–0.99) and non-fatal myocardial infarction (HR 0.74; 95% CI 0.51–1.08). Notably, semaglutide significantly improved HbA1c levels and reduced weight compared to placebo. The most common adverse events included nausea, vomiting and diarrhea [84]. In conclusion, the SUSTAIN-6 study demonstrated that semaglutide reduced the risk of MACE in patients with type-2 diabetes at high cardiovascular risk, improving glycemic control and promoting weight loss [85,86,87]. A more recent analysis of the SUSTAIN trial highlights the effects of GLP1-RA on kidney functions and cardiovascular outcomes in specific subgroups of patients, including those with different levels of estimated glomerular filtration rate (eGFR) and urinary albumin-to-creatinine ratio (UACR) [88]. The trial stratified patients into groups based on eGFR levels (normal kidney function, mild, moderate, or severe impairment); across eGFR subgroups, semaglutide demonstrated consistent efficacy in reducing HbA1c, body weight and cardiovascular events, irrespective of the baseline eGFR. Moreover, semaglutide reduced the decline in kidney function, particularly in patients with mild to moderate impairment [89]. In patients with more advanced kidney disease (lower eGFR), semaglutide maintained its glucose-lowering effect, although the decline in eGFR over time was less pronounced in these patients. In patients with reduced eGFR, semaglutide generally stabilized eGFR compared to placebo, with a slower rate of decline. Other analyses were performed on the effects of GLP1-RA on UACR subgroups. In brief, semaglutide caused a significant reduction in UACR, particularly in patients with higher baseline albuminuria (micro- and macroalbuminuria). These results suggest a protective effect of semaglutide in patients with kidney diseases. In patients with high UACR, semaglutide reduced albuminuria more significantly compared to placebo. These findings suggest that semaglutide could be used in a broad range of patients with type-2 diabetes, including those with chronic kidney diseases or albuminuria [89].

### 3.5. The SELECT Trial

The Semaglutide Effects on Cardiovascular Outcomes in People With Overweight or Obesity (SELECT) trial was a pivotal study aimed at demonstrating the cardioprotective properties of semaglutide in overweight or obese non-diabetic patients [90]. Participants included 17,604 adults with a body mass index (BMI) of 27 or greater (indicating overweight or obesity) and cardiovascular diseases. The primary aim was to evaluate the effectiveness of 2.4 mg of semaglutide (administered weekly via injection) in reducing MACE compared to a placebo [91,92]. The trial demonstrated for the first time that semaglutide reduced MACE by 20% [93]. Notably, participants experienced significant weight loss, which likely contributed to the cardiovascular benefits observed in this trial [94]. A recent analysis of the SELECT trial indicated the long-term benefits of semaglutide; in fact, patients experienced a lower incidence of death from kidney diseases and an improvement of estimated glomerular filtration rate (eGFR) compared to placebo. The cardiorenal benefits of semaglutide are due to its anti-inflammatory and anti-oxidative effects on podocytes of Bowman’s capsules [95]. 

### 3.6. STEP-HFpEF Trials

The STEP-HFpEF trials (Semaglutide Treatment Effect in People with Heart Failure with Preserved Ejection Fraction) evaluated the effects of semaglutide in patients with heart failure with preserved ejection fraction (HFpEF) [96]. HFpEF is well associated with metabolic comorbidities, including obesity and diabetes, and requires new preventive and therapeutic strategies [97]. Moreover, the STEP-HFpEF trial aimed to assess the impact of semaglutide on body weight and glycemic control in patient with HFpEF, and to evaluate its safety and tolerability [98,99,100,101]. Interestingly, patients treated with semaglutide (2.4 mg) showed improved quality of life and physical limitations and experienced weight loss compared to placebo. In another study called STEP-HFpEF DM (Research Study to Look at How Well Semaglutide Works in People Living With Heart Failure, Obe-sity and Type 2 Diabetes), among patients with obesity-related heart failure with preserved ejection fraction and T2DM, semaglutide reduced symptoms of HF and physical limitations; moreover, also in this case, patients showed reduced body weights compared to placebo [102,103].

Another interesting study analyzed the effects of semaglutide by sex across the STEP-HFpEF program: in a prespecified secondary analysis of pooled data from STEP-HFpEF and STEP-HFpEF DM, patients with HF, left ventricular ejection fraction ≥ 45%, body mass index ≥ 30 kg/m^2^, and KCCQ-CSS < 90 points were randomized 1:1 to once-weekly semaglutide 2.4 mg or matched placebo for 12 months. Women had a higher body mass index, left ventricular ejection fraction, C-reactive protein, and worse HF symptoms and coronary artery diseases vs. men [104]. Semaglutide improved KCCQ-CSS regardless of sex but reduced body weight in a more significant manner in women [104]. Notably, fewer serious adverse events were reported with semaglutide vs. placebo. In conclusion, the authors described that, in patients with obesity-related HFpEF, semaglutide 2.4 mg reduced body weight, especially in women, and it improved HF-related symptoms, physical limitations and exercise functions, regardless of gender [105] (Table 1). Importantly, these studies generally suggest that GLP-1 RA are effective in both genders in terms of glycemic control and weight loss, but there are some differences that may exist due to biological and hormonal factors [105]. GLP-1 RA consistently caused significant reductions in HbA1c levels for both men and women; however, the magnitude of the effect tended to be similar across the genders. Both genders experienced weight loss; however, women seemed to show better effects than men due to differences in fat distribution, hormonal responses and metabolism. Differences in estrogen levels could play a key role in the different degrees of responsiveness to GLP1RA between men and women [104,105]; in fact, estrogens may enhance the weight-reducing effects of GLP-1 RAs. 

### 3.7. PIONEER 6

The PIONEER 6 trial was a key cardiovascular outcomes study designed to assess the cardiovascular safety of oral semaglutide in patients with T2DM at high risk of cardiovascular events [106]. This trial was part of the larger PIONEER clinical program, which investigated the effectiveness and safety of oral semaglutide for use in the management of T2DM (Table 1). In more detail, PIONEER 6 was a randomized, double-blind, placebo-controlled, event-driven trial that enrolled 3183 patients with type-2 diabetes at high cardiovascular risk (e.g., with established cardiovascular disease, chronic kidney disease, or age ≥50 with cardiovascular risk factors); patients were randomly assigned to receive oral semaglutide (14 mg once daily) or placebo and followed for 16 months [107]. Notably, oral semaglutide was found to be non-inferior to placebo in terms of the incidence of MACE; specifically, MACE was 3.8% in the semaglutide group, compared to 4.8% in the placebo group (26% of risk reduction; HR: 0.79). A significant reduction in cardiovascular death in the semaglutide group was also seen (HR: 0.49 compared to placebo). No significant differences in non-fatal heart attacks or strokes between groups were seen. Importantly, semaglutide was associated with lower all-cause mortality (HR: 0.51).

The safety profile of oral semaglutide in PIONEER 6 was comparable to that shown in other trials with GLP1-RA [106]; common side effects included gastrointestinal events like nausea, vomiting, and diarrhea [108].

## 4. Discussion

Therapy with GLP-1 RA offers a new opportunity for the management of cardiovascular and metabolic risk in a wide range of patients, including those with/without T2DM and cancer [109,110,111]. However, some recent studies have indicated that GLP-1 RA could increase the risk of pancreatic cancer [112], but the evidence is not conclusive, and more recent large-scale studies and meta-analyses have provided mixed results. Moreover, recent preclinical studies in rodents showed an increased incidence of medullary thyroid carcinoma after therapy with GLP-1 RA [113], but no data in humans are actually available. On the contrary, other studies have indicated beneficial outcomes in cancer patients treated with GLP-1 RA, with anti-obesity effects and direct anti-cancer effects on cancer cells [114].

Cancer patients, particularly during chemotherapy or hormone therapy, require a cardiometabolic risk management program, which includes the prevention and treatment of hyperglycemia, visceral obesity and hypercholesterolemia [115]. GLP-1 RAs, due to their weight loss properties, may be beneficial in these patients, improving overall health and quality of life. Several cancer patients, especially those with pancreatic or colorectal cancer, may develop diabetes or hyperglycemia [116,117]; GLP-1 RA can provide effective glycemic control with a low risk of hypoglycemia, which is crucial for maintaining energy levels and overall health during cancer treatment [118]. A recent preclinical study suggested that GLP-1 RA might have anti-cancer properties, reducing cancer cell growth and inflammation in vitro through AMPK/mTOR and NLRP-3 pathways [118].

Cancer patients, especially long cancer survivors, are exposed to a high risk of several cardiovascular and metabolic diseases, including HF, arrhythmias, atherosclerosis, HFpEF, metabolic syndrome, T2DM, visceral obesity, insulin resistance, NAFLD, hypertension and venous thromboembolism [118,119,120,121]. For example, women with hormone-dependent breast cancer treated with aromatase inhibitors have a very high risk of metabolic syndrome, hypertension and visceral obesity, increasing the risk of cardiovascular mortality [122], and cardiometabolic diseases, through direct and indirect pathways, increased the risk of breast cancer recurrence [123]. Patients treated with immune check-point inhibitors (ICIs) are exposed to a three-times-higher risk of atherosclerosis. [124]. In most cases, ICIs are monoclonal antibody antagonists of programmed death-ligand 1 (PDL-1), programmed cell death protein 1 (PD-1) or cytotoxic T-Lymphocyte Antigen 4 (CTLA-4), which are the main drivers of peripheral immune tolerance, even towards tumors [125]. Briefly, ICIs antagonize the inhibition of lymphocyte uptake against tumors, resulting in a lymphocyte-mediated anticancer effect [126]. Recent trials associated ICIs with radiotherapy, standard chemotherapy (anthracyclines or platinum-based anticancer drugs), targeted therapies (HER-2 blocking agents, TKi and others) and combination therapies, i.e., PD-1 and CTLA-4 blocking agents [127]. In brief, combination therapies involving ICIs and standard chemotherapies increased lymphocytic infiltration in neoplastic tissue in a pro-inflammatory microenvironment, which makes CD56+ and CD3− large granular lymphocytes more reactive against tumor cells [128]. 

However, ICIs therapies are associated with a broad spectrum of endocrine diseases and cardiovascular events, including myocarditis, vasculitis, inflammatory endocrinopathies, mucositis and arthritis [129]. The main mechanisms of cardiotoxicity are not deeply understood, but NLRP-3/IL-1 overexpression, My-D88/TLR4, and cytokine-mediated pathways are still considered to be key orchestrators in ICIs-mediated side effects in preclinical and clinical models [130]. Very recently, atherosclerosis has emerged as a new considerable ICIs-mediated side effect in cancer patients through VCAM-1 and ICAM-1 overexpression in the luminal membrane of vascular endotheliocytes [131,132]. Therefore, GLP-1 RA therapy should be beneficial in ICIs-treated patients, reducing atherosclerotic events through direct and indirect effects (Figure 1 and Figure 2); first, GLP1-RA acts directly on the endothelial cells by reducing cholesterol accumulation, vascular inflammation, vascular smooth muscle activation and proliferation; on the contrary, GLP1-RA improves endothelial functions through the enhancement of nitric oxide production and ICAM-1/VCAM-1 expression [133,134]. The indirect effects of GLP-1 RA on atherosclerosis involve reductions in blood pressure, weight and glycated hemoglobin, which plays a key role in the pathogenesis of atherosclerosis [135].

## 5. Materials and Methods

A narrative search of the Medline and EMBASE databases was performed to identify potentially relevant papers reporting original research on the effects of GLP-1 RA in cancer patients. Preclinical and clinical studies on the beneficial effects of GLP-1 RA in cardiology and cardioncology published in English with available abstracts were selected if they addressed one or more of the following topics: GLP-1 receptor agonist, semaglutide, liraglutide, inflammation, diabetes, cardiotoxicity, cardio-protection and cancer. Reviews, case reports, and studies lacking immunomodulation activity endpoints or conducted in surgical settings were excluded. The following search strings were used: “GLP-1 receptor agonist AND cardiovascular diseases”, “GLP-1 receptor agonist AND cancer”, “GLP-1 receptor agonist OR semaglutide OR lariglutide AND cardiology”, “GLP-1 receptor agonist OR semaglutide OR lariglutide AND metabolism”, and “GLP-1 receptor agonist OR semaglutide OR lariglutide AND cardioncology”. The databases were last accessed on 10 August 2024.

## 6. Conclusions

Considering the promising cardiorenal outcomes of GLP-1 RA in patients with T2DM and cardiovascular diseases, a great cardioprotective potential could be assessed in cancer patients treated with cardiotoxic therapies. Cancer patients, especially long cancer survivors, are exposed to several cardio-metabolic diseases, including diabetes, heart failure and atherosclerosis, which increase their risk of cardiovascular mortality. This review sheds light on the biochemical properties of GLP-1 RA through a deep analysis of the clinical and preclinical evidence of the primary prevention of CTRCD. The systemic and cardiorenal benefits of GLP-1 RA support their use in cardio-oncology models, due to their anti-obesity and anti-inflammatory effects, driven by the activation of cAMP, PI3K/Akt and AMPK pathways and systemic increases in adiponectin and ET-1 levels. Therapy with GLP-1 RA reduced ferroptosis and systemic/myocardial levels of NLRP-3, MyD88, IL-1, IL-6 and IL-18, supporting their use in cancer patients treated with anthracyclines and ICIs. There is a pressing need to investigate anti-inflammatory and cardiorenal properties of GLP-1 RA in tumor-bearing models treated with highly cardiotoxic therapies (i.e., anthracyclines at total cumulative dose ≥ 400 mg/m^2^ alone or associated with radiotherapy or HER-2 blocking agents). The overall picture of the review encourages a study of the safety and efficacy of GLP-1 RA in cancer patients, especially in those at high or very high cardiovascular risk (i.e., history of CVD, T2DM, kidney disease, obesity) as a primary prevention strategy of HF and atherosclerosis.

## Figures and Tables

**Table 1:** A comprehensive summary table that includes results from key seven clinical trials of GLP-1 receptor agonists (GLP-1 RA) in cardiology, such as: LEADER, HARMONY Outcomes, EXSCEL, SUSTAIN-6, SELECT, STEP-HFpEF and PIONEER 6 trials. MACE includes cardiovascular death, non-fatal myocardial infarction, and non-fatal stroke.

Trial Name	GLP-1 RA	Primary Outcome	Duration	Results Summary
LEADER	Liraglutide	Major Adverse Cardiovascular Events (MACE)	3.5 years	Liraglutide significantly reduced MACE by 13% compared to placebo, demonstrating cardiovascular benefit.
HARMONY Outcomes	Albiglutide	MACE	1.6 years	Albiglutide reduced MACE by 22% compared to placebo, reflecting a positive cardiovascular benefit.
EXSCEL	Exenatide Extended Release	MACE	3.2 years	Exenatide did not significantly reduce MACE compared to placebo, but it did show trends towards cardiovascular benefits.
SUSTAIN-6	Semaglutide	MACE	2.1 years	Semaglutide showed a 26% reduction in MACE compared to placebo, indicating strong cardiovascular protection.
SELECT	Semaglutide (subcutaneous injection)	MACE	5.years	Semaglutide significantly reduced the risk of MACE of 20% compared to placebo. The trial met its primary endpoint, with fewer cardiovascular deaths, heart attacks, and strokes in the semaglutide group. The safety profile was consistent with previous studies.
STEP-HFpEF	Semaglutide (subcutaneous)	Change in Peak VO2 (exercise capacity)	1 year	Semaglutide significantly improved exercise capacity in patients with heart failure with preserved ejection fraction (HFpEF), though the impact on overall cardiovascular outcomes is still being assessed.
PIONEER 6	Oral Semaglutide	MACE	1.3 years	Oral Semaglutide demonstrated a 26% reduction in MACE compared to placebo, supporting its cardiovascular efficacy.

## Data Availability

Not applicable.
